# Utilization of High Iron Content Sludge and Ash as Partial Substitutes for Portland Cement

**DOI:** 10.3390/ma18102309

**Published:** 2025-05-15

**Authors:** Hui Gu, Zhenyong Zhang, Wen Li, Zhaobo Meng, Jianxiong Sheng

**Affiliations:** 1 School of Architecture and Engineering, Liaocheng University, Liaocheng 252000, China; zzy405308@163.com (Z.Z.); m19563562381@163.com (W.L.); mengzhaobo@lcu.edu.cn (Z.M.); 2 Wastewater Treatment Plant, Jia Ming Industrial Park, Liaocheng 252000, China; 13863435499@163.com

**Keywords:** sludge, sludge ash, supplementary cementitious material, compressive strengths, microstructure

## Abstract

Sludge is a semi-solid waste generated during the process of wastewater treatment. Due to the addition of polymerized ferric chloride in the flocculation process, the sludge produced by the sewage treatment plant in Liaocheng Jiaming Industrial Park contains a high content of iron oxide. In this paper, chemical analysis and particle size analysis of local sludge and sludge ash were conducted. In order to assess the potential of substituting cement as cementitious material with different dosages of sludge or sludge ash with high iron oxide content, setting time, compressive strength, microscopic analysis using microscopic testing (XRD, TG/DTG, SEM) and a toxicity characteristic leaching procedure (TCLP) were analyzed. These procedures determined the physical properties, compressive strength, hydration products, microstructure, and heavy metal contaminants of cement slurries replaced by local sludge or sludge ash with different dosages of high iron oxide content. The results show that less than 5% of local sludge or sludge ash can be incorporated into cement slurry as an alternative cementitious material for solid waste disposal.

## 1. Introduction

Sludge is a semi-solid byproduct generated during the process of wastewater treatment, primarily composed of organic matter, inorganic particles, microorganisms, water, and other components. Depending on its source and treatment methods, sludge can be classified into the following categories: municipal sludge, industrial sludge, water supply sludge, and dredged sludge. Current treatment methods for sludge in China include landfill, land utilization, natural drying, and incineration. Of these, 65% are landfill, 15% are land utilization, 6% are natural drying, and 3% are incineration [1,2,3]. Large quantities of sludge and sludge ash can be used as building materials for waste utilization. In EU countries, sludge reuse (e.g., composting and agricultural application) is the main method, 53% of sludge produced, followed by incineration (21%). After incineration, about 30% of the sludge remains as ash, which still contains heavy metals. A promising sustainable approach involves the incorporation of wastewater treatment byproducts in the production of building components, including low-density construction aggregates, bricks, and tiles [4]. Approximately 70 to 105 million tons of sewage sludge is generated worldwide each year [5]. To lower the carbon footprint of concrete production, the concrete industry has long promoted the partial replacement of Portland cement with supplementary cementitious materials (SCMs), including fly ash (FA) and ground-granulated blast furnace slag [6]. To guarantee complete decomposition of organic matter before application as an SCM in concrete, sewage sludge must undergo incineration at temperatures no lower than 700 °C [7]. Numerous studies have shown that sludge (SS) and sludge ash (SSA), as auxiliary cementitious materials, increase the setting time of cement pastes and reduce the strength and mechanical properties of cement solids [8,9,10,11,12,13]. Goh, C.C. [8], by blending SSA from the Senoko Incineration Plant into the net cement paste, found that the 28-day compressive strength increased by 9% at 5%, increased by 3% at 10%, decreased by 9% at 15%, and decreased by 21% at 20%. Chen, M. [9], by adding SSA incinerated in a fluidized bed incinerator into the cement paste, found that the 28-day compressive strength was reduced by 23% at 10%, 37% at 20%, 49% at 25%, and 51% at 30%. Naamane S [14] investigated the effects of sewage sludge treated at temperatures ranging between 300 °C and 800 °C on cement-based materials. The findings reveal that calcination alters the microstructure of SS, enhancing its pozzolanic activity, which peaks at 800 °C. Hui Gu [1] et al. pointed out that the content of amorphous phase in sludge incineration ash increases with the increase in calcination temperature. By comparing the compressive strength of mortar specimens made from sludge ash mixed with different calcination temperatures, it was found that the optimum calcination temperature for sludge incineration ash as an auxiliary cementitious material is 800 °C. Utilizing sludge or sludge ash as a partial cement replacement provides environmental advantages, including waste recycling and reduced CO_2_ emissions due to lower cement consumption. Additionally, these materials can enhance the mechanical performance of cementitious composites. Despite their potential, research on sludge and sludge ash applications remains limited.

The sludge from the sewage treatment plant in Liaocheng Jiaming Industrial Park, to which ferric chloride polymerization was added during the flocculation process, has a high iron oxide content in the resulting sludge. A comparison of the oxide composition of sludge and sludge ash calcined at 800 °C with that of cement is shown in Table 1. The SSA in the table was exposed to sunlight, then dried in a constant-temperature oven at 105 °C for 24 h, then calcined in a horse-boiling furnace at 800 °C for 6 h, and then taken out and cooled naturally [1]. As can be seen from the test results, the content of iron trioxide in SS and SSA is much greater than that in cement, reaching 58.31% and 54.83%, respectively. The high content of iron trioxide allows SS and SSA to act as fillers in the replacement of cement cementitious materials without hydration reactions. The silica and calcium oxide contained in SS and SSA are active in the substitution of cement cementitious materials and can undergo hydration reactions [15,16,17,18,19]. The phosphorus pentoxide and sulfur trioxide in them have the potential to cause an increase in the setting time of the slurry and a higher long-term compressive strength than the short-term compressive strength.

Based on this, this paper compares the potential of high iron oxide content SS and SSA as supplementary cementitious materials for cement, respectively. The feasibility of SS and SSA as supplementary cementitious materials was compared through setting time and 3-day and 28-day compressive strength tests on specimens with different dosages of SS and SSA. Analysis of hydration products and mechanisms of action of different dosages of SS and SSA as auxiliary cementitious materials was carried out by XRD and TG/DTG tests. Microstructural properties of SS and SSA as auxiliary cementitious materials with different dosages were obtained by scanning electron microscopy (SEM) analysis. Finally, the toxicity characteristic leaching procedure was used to analyze the curing effect of heavy metals when SS and SSA were used as auxiliary cementitious materials in different dosages. Then, the feasibility of SS and SSA with high iron oxide content as auxiliary cementitious materials was comparatively analyzed. The study in this paper provides a reference for the reuse of sludge generated in Kamin Industrial Park.

## 2. Materials and Methods

### 2.1. Materials

Shandong Province Liaocheng City Jiaming Sewage Treatment Plant, located west of Liaoling Road, covers an area of 70.5 acres. The total investment of the project is about 139 million yuan. The project is designed to treat 50,000 tons of sewage per day, with a construction scale of 20,000 tons/day for the first phase and 30,000 tons/day for the second phase. The project is mainly to deal with the Liaocheng Logistics Park, Jiaming Industrial Park, and the storage area of domestic sewage. Sewage treatment mainly comprises coarse and fine grating + lifting pumping station + fine grating + aeration sand sedimentation tank + biochemical tank + two sedimentation tank processes. The treatment also involves using the second lifting pumping station + magnetic sedimentation tank + ozone contact pool + a contact disinfection tank process. Solid wastes generated by the project are mainly sludge, slag, sand, and domestic garbage. Sludge is entrusted to a qualified unit for identification, and the identification of hazardous waste is entrusted to the appropriate category of hazardous waste treatment qualification units. After the identification of general solid waste, it is sent to the national environment of domestic waste incineration power plant for drying and incineration. Grate sludge, sunken sand, and domestic garbage are sent to a domestic garbage disposal site for landfill.

**Figure 1:**
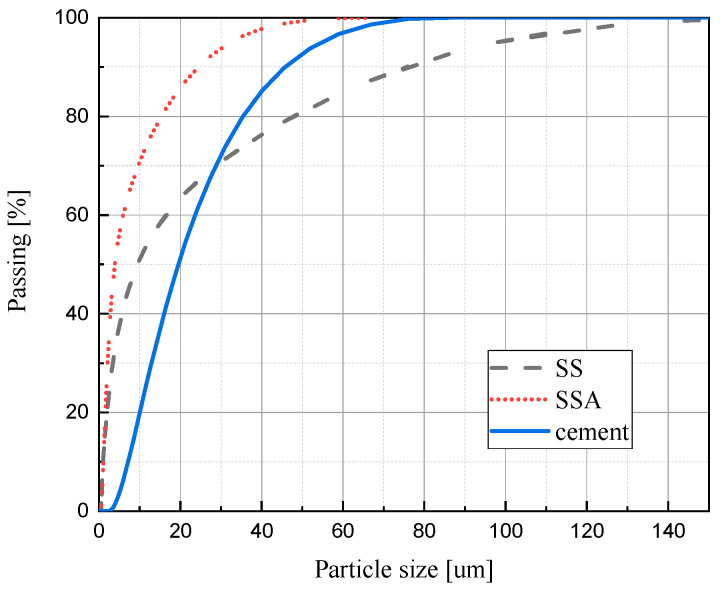
The cumulative particle size distribution of cement, SS, and SSA.

The sludge mentioned in this paper was taken from the sewage treatment plant of Liaocheng Jiaming Industrial Park, and the sludge and the incinerated sludge ash were ground with a 32,000 rpm FII-1000C mill (produced in Donghuancheng City, Guangdong Province, China), and the particle size distribution curves are shown in Figure 1. From the figure, it can be seen that the particle size distribution of SSA is smaller than that of cement, and the particle size distribution of SS is comparable to that of cement. The particle sizes of SSA and cement are more concentrated, and the particle sizes of SS are more dispersed. The D50 of cement, SS, and SSA are 19.5 um, 9.44 um, and 3.87 um. Smaller particle size can play a filling role in cement curing and is conducive to nucleation in cement hydration so that the hydration products are fixed in the nucleated material, increasing the strength of the cured material. The specific surface area of cement, SS, and SSA are 393.1 m^2^/kg, 1740 m^2^/kg, and 2185 m^2^/kg. The larger the specific surface area, the more water is required for hydration.

### 2.2. Mixture Design

The mixing ratios of mortars made with different dosages of SS and SSA as auxiliary cementitious materials are shown in Table 2. A fixed water–binder ratio (w/b) of 0.4 was used for all slurry–mortar systems. The control group used 100% pure cement. The SS dopings in SS-OPC were 0.1, 0.5, 1, and 5. The SS dopings in SSA-OPC were 0.5, 1, 5, 10, and 15. According to the previous literature review, the calcination temperature of sludge ash is taken as 800 °C. SS-10 was not included in the analysis here as it was found to be unable to be removed from the mold at 24 h during fabrication.

### 2.3. Sample Preparation and Characterizations

#### 2.3.1. Compressive Strength Study on Mortars

Mortars complying with EN 196 [20] were prepared according to the binder compositions specified in Table 2 to evaluate the development of compressive strength. The mixtures were poured into 40 mm × 40 mm × 40 mm molds, subjected to mechanical vibration for one minute, and then sealed with preservative film before being transferred to a controlled curing environment maintained at 20 ± 1 °C. After 24 h, the samples were removed from the molds and returned to the same curing conditions. For each mixture, six cement paste specimens were produced, and their compressive strength was measured after 3 and 28 days following ASTM C109 [21]. A Denison compression-testing machine was used for the assessments, applying a loading rate of 0.6 MPa/s with a maximum capacity of 3000 kN. The final results represent the mean values obtained from three tested specimens.

#### 2.3.2. Setting Time Test

The initial and final setting times of the cement paste were measured following ISO 9597:2008 [22] using a Vicat apparatus (Shanghai Luda Test Instrument Co., Ltd., Shanghai, China). The initial setting time was recorded when the needle penetration depth reached 34 ± 3 mm, while the final setting time corresponded to a penetration depth of 0.5 mm. To ensure accuracy, the final setting time was verified by conducting replicate tests at two additional locations on the sample.

#### 2.3.3. XRD, TG/DTG, and SEM

After demolding at 1 day, the specimens designated for XRD, TG/DSC, and SEM analysis underwent 28 days of curing in a controlled environment at 20 ± 1 °C. To examine hydration products, cement paste powders were prepared for XRD and TG/DTG analysis. At 28 days, the samples were crushed and immersed in isopropanol to arrest hydration. The fragments were then ground into fine powders and vacuum-dried at 40 °C for 24 h prior to testing. A Rigaku SmartLab (2025) 9 kW X-ray diffractometer equipped with a Cu-Kα radiation source (Kβ-filtered) was used for XRD analysis; a Bruker’s D8 ADVANCE X-ray diffractometer (Bruker D8 Advance, Bruker, Berlin, German) was also used. Scans were performed over a 2θ range of 5–65° at a scanning speed of 5°/min. Thermogravimetric analysis was conducted from 20 °C to 1100 °C with a heating rate of 10 °C/min. Both XRD and TG/DSC analyses were performed in a single run for each sample. Fractured cement paste fragments were analyzed using scanning electron microscopy to evaluate their surface morphology.

#### 2.3.4. Toxicity Characteristic Leaching Procedure (TCLP)

The leaching potential of heavy metals (HMs) from blended cement was evaluated using the toxicity characteristic leaching procedure (TCLP) on 28-day cured samples. The testing involved the following steps:

Sample preparation: Cement specimens were crushed to particles smaller than 9.5 mm and mixed with an acetic acid solution (pH 2.88) at a solid-to-liquid ratio of 1:20. Extraction process: The mixture was agitated in end-over-end rotating vessels at 30 rpm for 18 h to facilitate leaching. Leachate analysis: After filtration through a 0.45 μm membrane, the leachate was analyzed for trace elements (Ag, Cd, Cr, Cu, Pb, Zn, Ba, and Ni) via inductively coupled plasma atomic emission spectroscopy. ICP testing was performed using Perkin Elmer NexION 300D ICP-MS (Perkin Elmer, Springfield, IL, USA). Quality assurance: To ensure data reliability, triplicate extractions were performed, and the mean values were reported.

## 3. Results and Discussion

### 3.1. Setting Times

**Figure 2:**
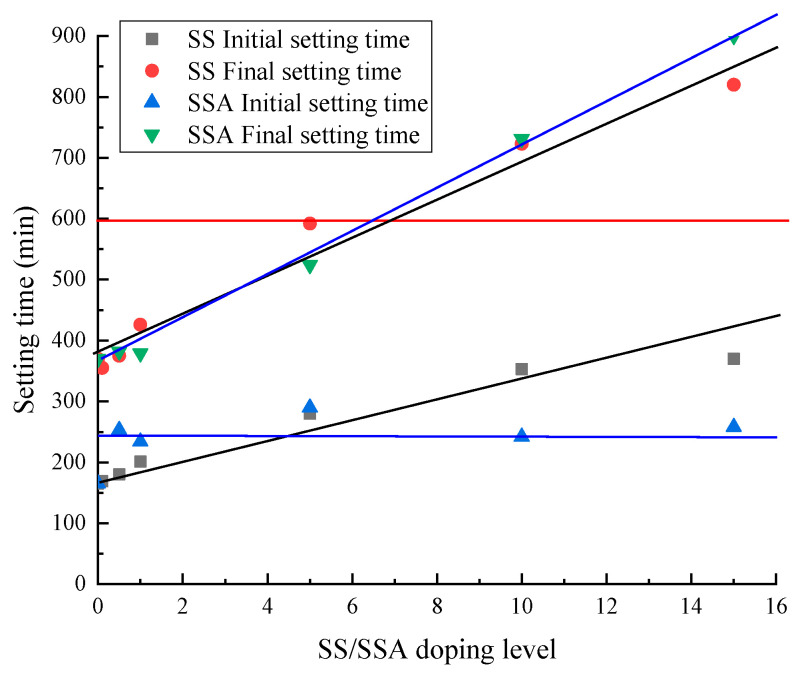
The setting times of cement pastes containing different contents of SS/SSA. The red line represents the specification GB/175-2007 in the final solidification time can not exceed 600 min of the provisions of the line. The black line represents the line where the initial and final setting time of SS increases with the doping amount. The blue line represents the line where the initial and final setting time of SSA increases with increasing dosage.

The current study surfaces that the incorporation of SS or SSA increases the setting time of the cement paste [23,24]. The plot of setting time of cement pastes with different SS or SSA dosages is shown in Figure 2. From the figure, it can be seen that both the initial setting time and final setting time of SS-doped mortar increased with the increase in SS dosage. The interval between the initial and final setting times maintains a good homogeneity. The final setting time of the mortar does not meet the specification of less than 10 h when SS is mixed at 10% and above, i.e., it is greater than 600 min [25]. The initial setting time of SSA-doped mortar is almost independent of the SSA dosage and is approximately distributed on a horizontal line. The final setting time of SSA-doped mortar almost linearly increased with the increase in SSA dosage. The final setting time of the mortar does not meet the specification of less than 10 h when SSA is mixed at 10% and above, i.e., it is greater than 600 min [25]. All combinations meet the specification GB/175-2007 [25] in the required initial setting time of more than 45 min. It can be seen that mortars mixed with less than 10% SS or SSA with high iron oxide content can meet the setting time requirements.

### 3.2. Compressive Strengths

**Figure 3:**
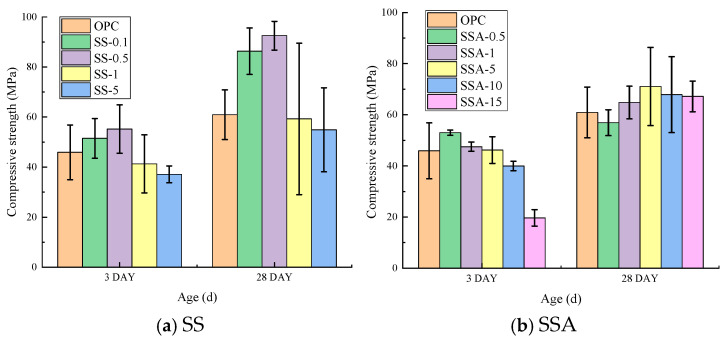
Compressive strength development containing different contents of SS/SSA.

The compressive strength development containing different contents of SS/SSA is shown in Figure 3. As can be seen from the figure, the 3-day and 28-day compressive strengths of SS at 0.1% and 0.5% doping were higher than that of the control mortar; the strength was comparable to that of the control mortar at 1% doping; the 3-day compressive strength decreased by 19.2% at 5% doping; and the 28-day compressive strength was comparable to that of the control mortar. The 3-day and 28-day compressive strengths of SS at 0.5%, 1%, and 5% doping were comparable to that of the control mortar; the 3-day compressive strength decreased by 12.9% and 57.1% at 10% and 15% doping, respectively, but the 28-day compressive strength was slightly higher than that of the control mortar. The 3-day and 28-day compressive strengths were comparable to those of the control mortar at 0.5%, 1%, and 5% SS dosages, while the 3-day compressive strengths were reduced by 12.9% and 57.1% at 10% and 15% dosages, respectively, and the 28-day compressive strengths were slightly higher than those of the control mortar. From the analysis results, it can be seen that less than 0.5% of SS can increase the compressive strength of the cementitious material due to its microfilling effect, and the long-term performance (28 days) of the structure is better; with a small amount of SSA, the compressive strength of the material is comparable to the strength of the mortar without sludge ash, and the short-term compressive strength significantly decreases when SSA is mixed with a larger amount, but its long-term compressive strength is unaffected and even higher than that of the control mortar.

### 3.3. Hydration Products

**Figure 4:**
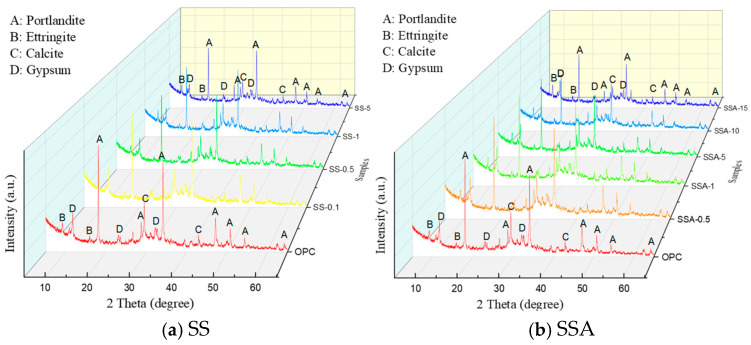
The 28-day XRD patterns of cement pastes incorporating varying amounts of SS or SSA.The different colored lines represent the XRD curves for different SS/SSA dopings.

XRD analysis was used to reveal the effect of high iron oxide content of SS or SSA on the hydration products of the cement paste. Figure 4 presents the 28-day XRD patterns of cement pastes incorporating varying amounts of SS or SSA. The samples were both qualitatively and quantitatively analyzed using the Jade software (MDI Jade 6). Jade is a powerful, full-featured powder XRD map-processing and analysis software, especially focusing on quantitative analysis and identification of physical phases, which can realize the accurate identification and quantitative analysis of unconventional small amounts of physical phases. The crystalline phases and chemical composition contain portlandite (Ca(OH)_2_), ettringite (Ca_6_Al_2_(SO_4_)_3_(OH)_12_•26H_2_O), calcite (CaCO_3_), and gypsum (CaSO_4_•2H_2_O). Portlandite peaks are the most abundant. The physical phases of slurries with different dosages of SS and SSA are almost identical to those of pure cement slurries after hydration at 28 days. This is the main reason for the similarity in the 28-day compressive strength between different dosages of SS or SSA mortar and pure cement mortar. A small amount of SS or SSA has less effect on the hydration products of mortar. Due to the high iron oxide content in SS and SSA, the product also contains a small amount of calcium iron oxide (Ca_2_Fe_9_O_13_). Not labeled in the figure due to its low content.

**Figure 5:**
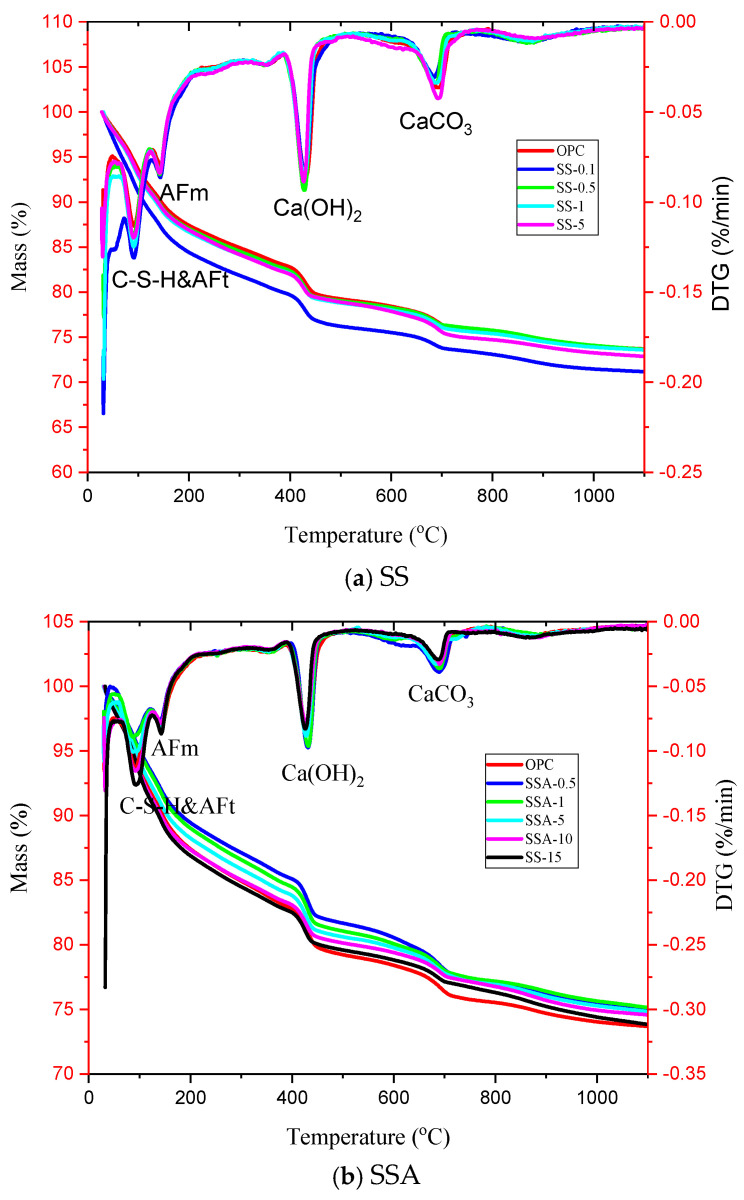
TG-DTG curves for cement pastes incorporating varying amounts of SS or SSA.

The hydration products were quantified based on thermogravimetric and derivative thermogravimetry (TG-DTG) data. Figure 5 presents the TG-DTG curves for both ordinary Portland cement (OPC) and cement mixtures containing different proportions of SS or SSA. The thermal stability of samples was measured by a HITACHI STA200 simultaneous thermal analyzer with a heating rate of 10 °C/min from room temperature to 1100 °C in an N_2_ atmosphere. The TG and DTG curves at 28 days for pure cement and cement paste with different SS and SSA dosages in Figure 5 are similar, with four exothermic peaks. The DTG (derivative thermogravimetry) curves of cement pastes containing different proportions of SS or SSA after 28 days of curing exhibit four distinct endothermic peaks. The first peak, occurring between 100 and 150 °C, is primarily attributed to the breakdown of C-S-H and AFt. A second thermal event appears in the range of 160–190 °C, corresponding to the dehydration of AFm. The third peak, observed at approximately 400–450 °C, arises from the thermal decomposition of calcium hydroxide (Ca(OH)_2_). Additionally, a fourth endothermic reaction occurs at 650–720 °C, indicating the decarbonation of crystalline calcium carbonate (CaCO_3_). Since the 28-day hydration exothermic peaks of the mortars containing different dosages of SS and SSA in the figure are similar to those of the pure mortar, their 28-day compressive strengths are close to each other, which is in line with the results of the compressive strength tests.

### 3.4. Microstructure

**Figure 6:**
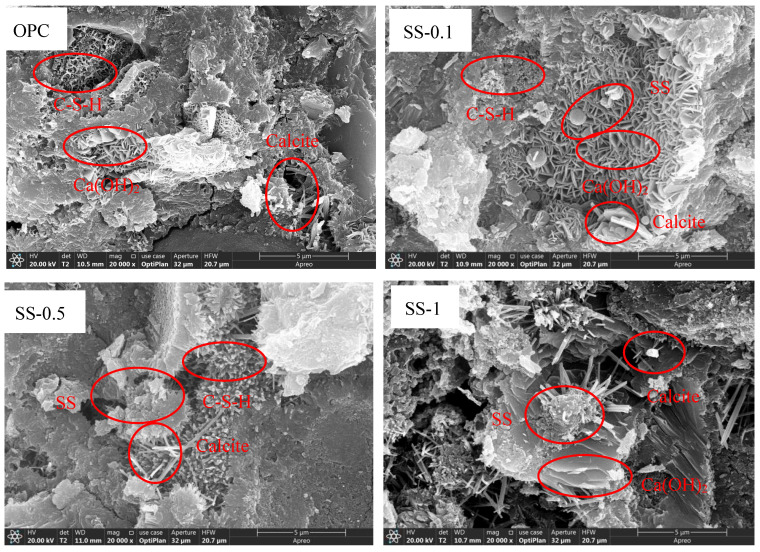

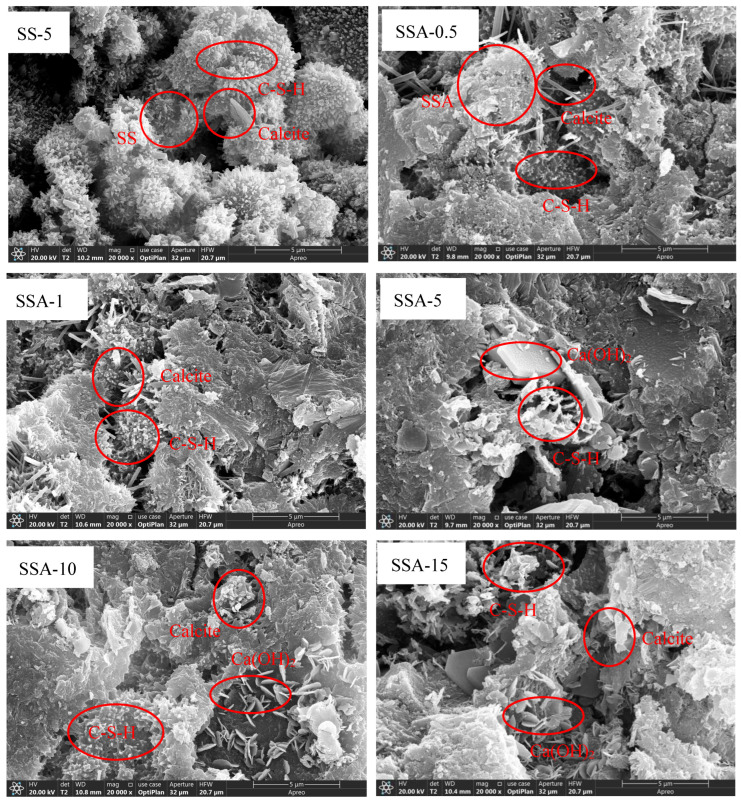
The surface morphology of cement composites incorporating different proportions of SS and SSA.

The surface morphology of cement composites incorporating different proportions of SS and SSA was investigated using scanning electron microscopy (SEM), with primary focus on hydration products and pore structure analysis. Comparative microstructural images of the SS and SSA-modified specimens at 28 days are displayed in Figure 6. As can be seen from the figure, the main hydration products are C-S-H, Ca(OH)_2_, and calcite, consistent with the results of XRD and TG analyses. Bead-like sludge particles can be seen in the cementitious sand with a lower dosage of SS. Some of the hydration products form a dense structure with the sludge as the nucleus, which is conducive to the enhancement of the compressive strength of the material. At SS additions of 0.1% and 0.5%, the glued sand specimens produced a matrix material with high density and good surface properties. These results are consistent with the low water absorption, high compressive strength, and high bulk density of the specimens. SS-0.1 and SS-0.5 have a denser microstructure compared to the other groups, and therefore their tested 28-day compressive strengths are higher. The microstructures of the other SS and SSA added materials showed similarly dense matrix materials. However, larger pores appeared in these specimens, and the products also had relatively low bulk densities and high compressive strengths. Some SS and SSA do not hydrate in cement cementitious materials but act as fillers, and their presence can be directly observed in the microstructure. The results were consistent with previous studies, i.e., the 28-day compressive strength of the specimens with 0.1% and 0.5% SS was higher than that of the control group, and the compressive strength of the other specimens was comparable to that of the control group.

### 3.5. Environmental Properties

The raw SSA employed in this investigation exhibited varying levels of metal(loid)s, as detailed in prior research [1]. According to that study, Zn and Cu were the most abundant elements, with significant quantities of Sr, Cr, and Ba also present. Additionally, TCLP analysis revealed that the leachable fractions of these metal(loid)s were substantially lower than the permissible thresholds set by TCLP regulations.

The dissolved concentrations of heavy metals in cement mortars with different SS/SSA dosages are given in Table 3. For mortar samples with different SS or SSA dosages, the dissolved concentrations of different metals(loid)s were below the specified TCLP limits and GB 5085.3-2007 [26] limits. The metals(loid)s dissolved from the mortar specimens were effectively immobilized, indicating that the metals(loid)s were tightly bound after hydration of the cementitious material, and the harmful effects on the environment were minimal. Therefore, cement mortars made with a certain amount of SS or SSA do not cause environmental and health problems.

## 4. Conclusions

The feasibility study of using sludge and sludge ash with high iron trioxide content from the sewage treatment plant of Liaocheng Jiaming Industrial Park as alternative cementitious materials and blending them into cement in a suitable proportion was conducted to compare the role mechanism and application potential of SS and SSA with high iron oxide content as auxiliary cementitious materials for cement. The mechanism of action and application potential of SS and SSA with high iron oxide content as supplementary cementitious materials were compared, and the following conclusions were obtained:

(1) The mixing of SS and SSA will prolong the setting time of the cementitious material, and the setting time of the cementitious material meets the requirements of the national standard GB/175-2007 when the mixing amount does not exceed 5%.
(2) The compressive strength of mortar doped with not more than 1% SS has some improvement, and the compressive strength of SS- and SSA-doped with not more than 5% is comparable to that of the control mortar. The long-term performance of SS- and SSA-doped cementitious materials is better than the short-term performance. At low dosage, it is more economical to directly mix SS.
(3) The hydration products of mortars with different dosages of SS and SSA were the same at 28 days. XRD analysis showed that the crystalline phases and chemical composition contain portlandite, ettringite, calcite, and gypsum. TG-DTG analysis showed four exothermic peaks: C-S-H and AFt, AFm, Ca(OH)_2_, and CaCO_3_. SEM showed a denser microstructure for mortars doped with up to 1% SS. SS and SSA with high iron content are partly involved in hydration and partly used as filler materials in the pores in cement mortar, and the microstructure is denser when SS and SSA are doped up to 5%.
(4) For mortar samples with different SS or SSA dosages, the dissolved concentrations of heavy metals were below the specified TCLP limits and GB 5085.3-2007 limits. The mortar samples with low dosages of SS and SSA were not harmful to the environment.
(5) Sludge and sludge ash from the sewage treatment plant of Liaocheng Jiaming Industrial Park with high ferric oxide content, at dosages less than 5%, are feasible as auxiliary cementitious materials for cement.

## Figures and Tables

**Table 1:** Chemical composition of the cement, SS, and SSA.

Material	Chemical Composition (%)								
	SiO_2_	CaO	Al_2_O_3_	Fe_2_O_3_	MgO	Na_2_O	K_2_O	P_2_O_5_	SO_3_
Cement	25.48	54.45	7.89	4.35	1.4	0.6	0.64	0.15	4.12
SS	14.67	3.23	4.05	58.31	1.17	1.71	0.97	9.04	3.1
SSA	16.06	3.61	4.98	54.83	1.23	1.41	1.08	11.97	2.23

**Table 2:** Detailed mixture proportions of OPC, SS-OPC, and SSA-OPC systems studied (wt%).

System	w/b	Cement	SS	SSA	
				Proportions	Calcination Temperature
OPC	0.4	100	0	/	/
SS-0.1	0.4	99.9	0.1		
SS-0.5	0.4	99.5	0.5		
SS-1	0.4	99	1	/	/
SS-5	0.4	95	5	/	/
SSA-0.5	0.4	99.5	/	0.5	800
SSA-1	0.4	99	/	1	800
SSA-5	0.4	95	/	5	800
SSA-10	0.4	90	/	10	800
SSA-15	0.4	85	/	15	800

**Table 3:** TCLP leaching concentrations from cement paste with different SS/SSA dosages.

Application	Metal(loid) (mg/L)							
	Ag	Cd	Cr	Cu	Pb	Zn	Ba	Ni
SS-0.1	ND	ND	0.0010	0.0062	0.0053	0.0020	2.7913	0.0027
SS-0.5	ND	ND	0.0037	0.0318	0.0053	0.0031	3.0953	0.0106
SS-1	ND	ND	0.0014	0.0733	0.0056	0.0033	2.9892	0.0260
SS-5	ND	ND	0.0038	0.1007	0.0062	0.0056	2.2478	0.0582
SSA-0.5	0.0001	ND	0.0044	0.0015	0.0032	0.0031	2.9940	0.0008
SSA-1	ND	ND	0.0034	0.0014	0.0036	0.0028	3.0494	0.0008
SSA-5	ND	ND	0.0148	0.0012	0.0035	0.0043	2.8374	0.0008
SSA-10	ND	ND	0.0139	0.0029	0.0032	0.0049	2.6573	0.0010
SSA-15	ND	ND	0.0077	0.0012	0.0040	0.0033	2.4074	0.0008
TCLP ^a^ regulatory limit	5	1	5	15	5	25	100	25
GB 5085.3-2007 ^b^ limit	5	1	15	100	5	100	100	5

ND: not detected; ^a^ TCLP: toxicity characteristic leaching procedure; ^b^ Chinese Standard: Identification Standards for Hazardous Wastes-Identification for extraction toxicity.

## Data Availability

The original contributions presented in this study are included in the article. Further inquiries can be directed to the corresponding author.
